# Lower human defensin 5 in elderly people compared to middle-aged is associated with differences in the intestinal microbiota composition: the DOSANCO Health Study

**DOI:** 10.1007/s11357-021-00398-y

**Published:** 2021-06-08

**Authors:** Yu Shimizu, Kiminori Nakamura, Mani Kikuchi, Shigekazu Ukawa, Koshi Nakamura, Emiko Okada, Akihiro Imae, Takafumi Nakagawa, Ryodai Yamamura, Akiko Tamakoshi, Tokiyoshi Ayabe

**Affiliations:** 1 Department of Cell Biological Science, Faculty of Advanced Life Science, Hokkaido University, North 21, West 11, Kita-ku, Sapporo, Hokkaido 001-0021 Japan; 2 Reserch Unit of Advanced Interdisciplinary Care Science, Osaka City University Graduate School of Human Life Science, 3-3-138, Sugimoto, Sumiyoshi-ku, Osaka, 558-8585 Japan; 3 Department of Public Health and Hygiene, Graduate School of Medicine, University of the Ryukyus, 207, Uehara, Nishihara-cho, Okinawa, 903-0215 Japan; 4 Department of Nutritional Epidemiology and Shokuiku, National Institute of Biomedical Innovation, Health and Nutrition, 1-23-1, Toyama, Shinjuku-ku, Tokyo, 162-8636 Japan; 5 Suttu Municipal Clinic, 72-2, Toshimacho, Suttu-cho, Hokkaido 048-0406 Japan; 6 The Centre of Family Medicine, 1-18, North 41, East 15, Higashi-ku, Sapporo, Hokkaido 007-0841 Japan; 7 Division of Biomedical Oncology, Institute for Genetic Medicine, Hokkaido University, North 15, West 7, Kita-ku, Sapporo, Hokkaido 060-0815 Japan; 8 Department of Public Health, Faculty of Medicine, Hokkaido University, North 15, West 7, Kita-ku, Sapporo, Hokkaido 060-8638 Japan

**Keywords:** α-Defensin, Human defensin 5, Paneth cells, Intestinal microbiota, Immunosenescence, Aging

## Abstract

Recently, aging is considered a risk factor for various diseases. Although changes in the intestinal microbiota along with aging are thought to associate with the increased disease risk, mechanisms that cause age-related transition of the intestinal microbiota remain unknown. This study aims to clarify relationships between the amount of human defensin 5 (HD5), a Paneth cell α-defensin, which is known to regulate the intestinal microbiota, and age-related differences of the intestinal microbiota composition. Fecal samples from 196 healthy Japanese (35 to 81 years old) were collected and measured HD5 concentration. HD5 concentration in the elderly group (age > 70 years old) was significantly lower than the middle-aged group (age ≤ 70 years old). Furthermore, individual age was negatively correlated with HD5 concentration (*r* =  − 0.307, *p* < 0.001). In β-diversity, the intestinal microbiota of the elderly showed a significantly different composition compared to the middle-aged. At the genus level, relative abundance of *Collinsella*, *Alistipes*, Peptococcaceae; unassigned, *Lactobacillus*, *Lactococcus*, *Weissella*, *Christensenellaceae R-7 group*, *Megasphaera*, and *[Eubacterium] eligens group* was significantly higher, and Lachnospiraceae; unassigned, *Blautia*, *Anaerostipes*, *Fusicatenibacter*, *Dorea*, and *Faecalibacterium* was significantly lower in the elderly compared to the middle-aged. In addition, HD5 concentration was negatively correlated with *Alistipes*, Peptococcaceae; unassigned, and *Christensenellaceae R-7 group* and positively correlated with Lachnospiraceae; unassigned and *Dorea*. These results provide novel insights into the immunosenescence of enteric innate immunity, indicating low HD5 is suggested to contribute to the age-related differences in the intestinal microbiota and may relate to increased risk of diseases in elderly people.

**Supplementary Information:**

The online version contains supplementary material available at 10.1007/s11357-021-00398-y.

## Introduction

A rapid increase of aged people, aging, has been progressing all over the world. According to the United Nations, the world population of people aged 65 years old and over, which was 0.73 billion in 2019, is estimated to 1.5 billion by 2050 [1]. Epidemiological studies showed that aging is a risk factor for the onset or severity of various diseases such as cardiovascular diseases, dementia, cancer, neurodegenerative diseases, infectious diseases, diabetes, and non-alcoholic fatty liver disease (NAFLD) [2–5], indicating that aging is considered as a global issue in public health.

It has been known that aging is accompanied by the downregulation in immune function, termed immunosenescence characterized by features such as reduction in phagocytotic activities of neutrophils and macrophages, impaired reactivities against bacterial antigens of monocytes in peripheral blood, and decrease in the number of naïve lymphoid cells [6]. The intestine is the largest immune organ [7] and harbors about 40 trillion bacteria of the intestinal microbiota, forming a complex ecosystem in humans [8]. The human intestinal microbiota is estimated to possess 10 million genes [9] and involved in various host physiological functions including assimilation of dietary fibers [10], vitamin synthesis [11], metabolism of secondary bile acids [12], regulation of immune cell differentiation [13, 14], and nervous system development [15]. Recently, relationships between the imbalance of the intestinal microbiota, dysbiosis, and many diseases including Crohn’s disease [16], obesity [17], diabetes [18], graft versus host disease [19], cancer [20], and Alzheimer’s disease [21] have been reported. Because the intestinal microbiota composition changes along with aging [22, 23], relationships between the intestinal microbiota and immunosenescence along with aging have been suggested [24]. In addition, studies using germ-free animal models showed that altered intestinal microbiota along with aging induces increased intestinal permeability, systemic inflammation, and decreased cognitive function [25, 26]. Thus, it has been considered that compositional changes in the intestinal microbiota associates with the increase of disease risk along with aging.

The intestinal epithelium which contacts with the intestinal microbiota is known to play important roles not only as the first line of defense against pathogens but also in regulation of the intestinal microbiota composition [27]. Paneth cells, a lineage of terminally differentiated epithelial cells located in the base of the small intestinal crypts, regulate regeneration and differentiation of small intestinal epithelium by constituting a stem cell niche [28]. Paneth cells have intracellular secretory granules rich in antimicrobial peptide α-defensins termed cryptdins (Crps) in mice [29, 30] and human defensin (HD) 5 and 6 in humans [31, 32], and contribute to innate enteric immunity by secreting α-defensins into the intestinal lumen in response to bacterial stimuli [33, 34], cholinergic agents [35], and certain food factors [36]. Furthermore, Paneth cell α-defensins have been known to contribute to the regulation of the intestinal microbiota in their quantity and quality-dependent manners [37–42]. It has also been reported that the supply of Wnt signals to the stem cells by Paneth cells is diminished by aging [43, 44], suggesting that Paneth cell function declines with aging. Taken together, functional deficiencies of Paneth cell α-defensins along with aging may induce the transition of the intestinal microbiota composition and further relate to increased risk of diseases in the elderly. However, whether aging affects the secretion of Paneth cell α-defensins remains unknown. We aim in this study to elucidate the relationship between amounts of HD5 secretion and compositional changes in the intestinal microbiota along with aging by analyzing fecal samples of 196 healthy Japanese having no medical treatments who joined the Dynamics of Lifestyle and Neighborhoods Community on Health Study (the DOSANCO Health study), a community-based study conducted in the town of Suttu, Hokkaido [45]. Here we show that the amount of fecal HD5 is lower in the elderly people compared to middle-aged and the low HD5 correlates with the age-related differences in the intestinal microbiota, possibly suggesting Paneth cell α-defensin-dependent immunosenescence.

## Methods

### Study design and population

Data and fecal samples used in this study were obtained as part of the Dynamics of Lifestyle and Neighborhood Community on Health Study (the DOSANCO Health Study), a population-based cohort study conducted in the town of Suttu, Hokkaido, Japan, during 2015 [45]. Briefly, 2100 participants (977 males and 1123 females) comprising 79.6% of all residents who were 3 years old or older, and who have not lived in nursing homes, responded to a self-administered questionnaire. For participants of elementary school age or under, their parents filled out the questionnaire instead. The questionnaire collected information about age, gender, medical history, and lifestyle. Of the 2100 participants, 629 complied with a request for providing fecal samples. Participants themselves collected their fresh fecal samples with collection kits and packed into a cooler bag with frozen refrigerants and brought it to the place where the health checkup was conducted. Samples were frozen immediately after collected by researchers at – 30 °C, then transported on dry ice to the laboratory, and stored at – 80 °C until using. Of these 629 participants, 331 participants with enough amounts of fecal samples were included in both quantifications of HD5 concentration and 16S rDNA sequencing. From these 331 participants, 135 participants were excluded due to undergoing clinical treatment of diabetes, gastric ulcer, duodenal ulcer, hepatitis, liver cirrhosis, and other digestive system diseases which may directly influence the intestinal environment (n = 74), insufficient data quality of 16S rDNA sequencing (n = 61, detailed criteria are denoted in the part of 16S rDNA-based taxonomic analysis in the Methods section). Consequently, data from 196 participants were analyzed. In this study, we defined participants aged 70 years old and younger as the middle-aged group (n = 132) and after 70 years old as the elderly group (n = 64) based on the survey conducted by the Cabinet Office of the Japanese Government showing that most Japanese think that people aged 70 years old and over should be considered as elderly [46]. The study design was approved by the Ethical Committee of the Faculty of Medicine (15–002, 15–045), Hokkaido University. Written informed consent was obtained from all participants.

### Preparation of oxidized-form HD5

Chemically synthesized HD5 (Thermo Fisher Scientific, Waltham, MA) was dissolved in water containing 3 mM reduced glutathione, 0.3 mM oxidized glutathione, and 8 M urea. The solution was adjusted to pH 8.4 by adding 0.25 M NaHCO_3_; then, oxidative folding was conducted by gently mixing at room temperature for 18 h. Oxidized-form HD5 was purified by reverse-phase high-performance liquid chromatography as described previously [47].

### Production and screening of monoclonal antibodies against oxidized-form HD5

C3H/HeJJmsSlc-lpr/lpr mice (Japan SLC, Shizuoka, Japan) were injected intraperitoneally with an emulsion containing 0.2 mg of oxidized-form HD5 and Freund’s complete adjuvant. After 2 weeks, an emulsion containing 0.2 mg of oxidized-form HD5 and Freund’s incomplete adjuvant. Two weeks later, mice were injected intraperitoneally with 0.2 mg of oxidized-form HD5 dissolved in phosphate-buffered saline (PBS) as the last booster injection. After the final injection, mice were euthanized and splenocytes were obtained. Hybridomas were produced by fusing the splenocytes with mouse myeloma P3U1 cells in the presence of 50% polyethylene glycol (Sigma-Aldrich, St. Louis, MO). Hybridoma clones producing oxidized-form HD5-specific antibody were screened by indirect enzyme-linked immunosorbent assay (ELISA) as described previously [47] and subcloned four times by the limiting dilution method. Reactivities of each clone pair against oxidized-form HD5 were evaluated by comparing the sensitivities of sandwich ELISA, and the pair of clones 12-1 and 39E-7 showing the highest sensitivity against oxidized-form HD5 was finally selected for the establishment of sandwich ELISA.

### Extraction of fecal proteins

Fecal samples were dried by lyophilization and pulverized to powder using a beads beater-type homogenizer (Multi-beads shocker; Yasui Kikai, Osaka, Japan). Ten mg of fecal powder was suspended with 100 μL of PBS (-) and voltex mixed at 4 °C overnight. Precipitates were removed by centrifugation at 4 °C, 15,000 g for 30 min.

### Quantification of fecal HD5 by sandwich ELISA

As standards for quantification of fecal HD5, oxidized-form HD5 was dissolved in PBS (-). Capture antibody (12-1) was dissolved in 50 mM sodium carbonate-bicarbonate buffer (pH 9.6) at a concentration of 1 mg/mL. Biotinylated detection antibody (39E-7) was dissolved in 50 mM sodium carbonate-bicarbonate buffer at a concentration of 0.5 mg/mL. One hundred microliters of the capture antibody solution was added to each well of 96 well microtiter plate, then incubated at 4 °C overnight. After the incubation, the plate was washed by PBS with Tween 20 (PBS-T) for removing excess antibody; then, each well was blocked by 200 μL of 25% Block Ace (DS Pharma Biomedical, Osaka, Japan) at 25 °C for 1 h. After removing the excess amount of blocking solution, 100 μL of standards or fecal extracts were added to the well and reacted with a capture antibody at 25 °C for 1 h. After washing by PBS-T, 100 μL of detection antibody solution was added to each well and incubated at 25 °C for 1 h. Subsequently, 100 μL of streptavidin–horseradish peroxidase conjugate (GE Healthcare, Piscataway, NJ) in a 1:5000 dilution was added to each well and incubated at 25 °C for 1 h. After washing, 100 μL of tetramethylbenzidine (TMB) chromogen substrate buffer (SureBlue; Kirkegaard & Perry Laboratories, Gaithersburg, MD) was added to each well and incubated at 25 °C for 30 min. To stop the color reaction of TMB, 100 μL of 0.6 N H_2_SO_4_ was added, and absorbance values were measured at 450 nm using a microplate reader (Multiscan FC; Thermo Fisher Scientific).

### Purification of fecal DNA

Total genomic DNA was extracted and purified from 200-mg fecal samples using QIAamp Fast DNA Stool Mini Kit (QIAGEN, Hilden, Germany) following the manufacturer’s protocol. Final DNA concentrations were determined based on 260 nm absorbance using a Nanodrop 2000 spectrometer (Thermo Fischer Scientific).

### 16S ribosomal DNA (rDNA) sequencing

Amplification of 16S rDNA in each fecal DNA sample was conducted by polymerase chain reaction (PCR) using a universal primer set of Bakt 341F (5-cctacgggnggcwgcag) and Bakt 805R (5-gactachvgggtatctaatcc) which covers the V3-V4 variable region of 16S rDNA [48]. First PCR amplification was conducted in 25 μL of reaction mixtures containing 12.5 ng of fecal DNA, 200 nM of each primer, and 1 × KAPA HiFi Hot Start Ready Mix (Kapa Biosystems, Wilmington, MA) under the following conditions: 95 °C for 3 min, 25 cycles of 95 °C for 30 s, 55 °C for 30 s, and 72 °C for 30 s, followed by 72 °C for 5 min. First PCR products were purified with AMPure XP beads (Beckman Coulter, Brea, CA). After purification, second PCR was conducted for adding sequencing adapters containing sample-specific 8-bp barcodes to the 3’- and 5’-ends of the first PCR products by using the Nextera XT Index Kit v2 Set B (Illumina, San Diego, CA). Second PCR amplification was conducted in 50 μL of reaction mixtures containing 5 μL of first PCR amplicon, 5 μL of each indexing primer, and 1 × KAPA HiFi Hot Start Ready Mix under the following conditions: 95 °C for 3 min, 8 cycles of 95 °C for 30 s, 55 °C for 30 s, and 72 °C for 30 s, followed by 72 °C for 5 min. Each amplicon was purified, quantified using the Qubit dsDNA HS Assay Kit (Thermo Fischer Scientific), and then adjusted to 4 nM. Second PCR Amplicons were pooled 4 μL each and subjected to quantification using KAPA Library Quantification Kit Lightcycler 480 qPCR Mix (Kapa Biosystems) and then diluted to 4 pM. The amplicon library was combined with 5% equimolar PhiX Control v3 (Illumina) and sequenced on a MiSeq instrument using the MiSeq 600-cycle v3 kit (Illumina) by pair-end sequencing mode.

### 16S rDNA-based taxonomic analysis

Demultiplexed pair-end sequencing reads were imported into the QIIME2 pipeline (version 2019.7) [49]. Imported sequences were quality filtered and denoised, and chimeric sequences were removed by DADA2 plugin [50] with following parameters; –p-trim-left-f 17, –p-trim-left-r 21, –p-trunc-len-f 280, –p-trunc-len-r 200, –p-max-ee-f 2 –p-max-ee-r 2. In this step, samples containing chimeric sequences of more than 50% were excluded from this study because the data quality of the 16S rDNA sequencing was considered insufficient for the analysis. The phylogenic tree was calculated and rooted with FastTree [51] after alignment with MAFFT [52]. Taxonomic assignments of each feature were conducted using a naïve Bayes classifier trained on 16S rRNA gene OTUs clustered at 99% similarities within the Silva database (v132). Using QIIME2 workflow, α-diversity (Simpson index) and β-diversity (unweighted UniFrac distance) were estimated. Statistical significance of β-diversity was determined by Permutational multivariant analysis of variance (PERMANOVA) test in QIIME2 pipeline.

### Statistical analysis

All statistical analyses were conducted by Prism ver. 7.0 software (GraphPad, San Diego, CA). Statistical significance was determined by a two-sided unpaired Student’s *t*-test between two groups, and correlation analyses were performed by Pearson’s correlation coefficients test. In all statistical tests, *p* < 0.05 was considered statistically significant. If not stated otherwise, data are shown as mean ± standard deviation (SD).

## Results

### Amounts of secreted human defensin 5 in the elderly people is lower than middle-aged

**Fig. 1:**
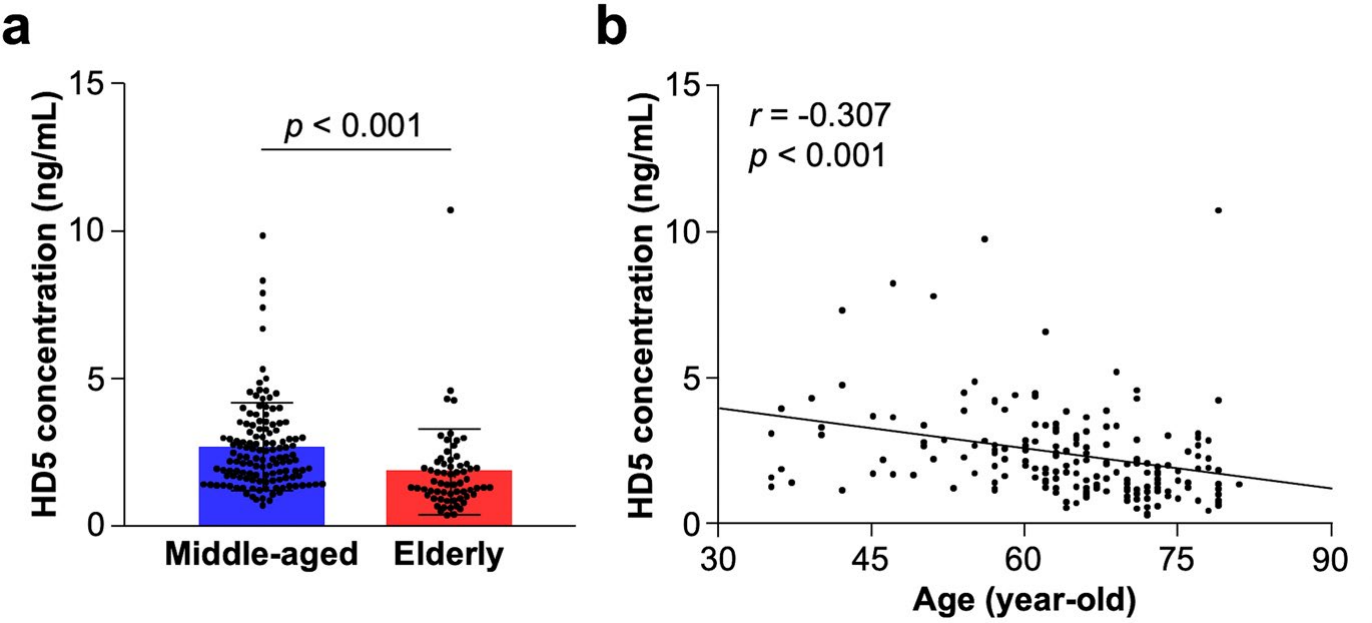
Secretory amount of HD5 in elderly people is lower than middle-aged. **a** Comparison of fecal HD5 concentration between middle-aged and elderly group. **b** Correlation analysis between fecal HD5 concentration and age in all participants. Error bars represent mean ± SD. Statistical significance was evaluated by unpaired Student’s *t*-test in **a** and Pearson’s correlation coefficients test in **b**

First, to analyze the relationship between the amount of HD5 secretion and aging, fecal HD5 concentration measured by sandwich ELISA was compared between middle-aged (age ≤ 70 years old) and elderly group (age > 70 years old). General information of participants in each group is shown in Table 1. Fecal HD5 concentration in the elderly was significantly lower than that in the middle-aged (Fig. 1a, middle-aged vs elderly: 2.58 ± 1.51 vs 1.82 ± 1.47 ng/mL, *p* < 0.001). Furthermore, a significant negative correlation was observed between fecal HD5 concentration and age in all participants (Fig. 1b, r =  − 0.307, *p* < 0.001). There was no significant difference in fecal HD5 concentration between male and female participants (Supplementary Fig. 1). These results indicate that the elderly people show lower secretion of HD5 compared to the middle-aged.

**Table 1:** General information of participants

Number of participants (male/female)	
All participants	196 (89/107)
Middle-aged (age ≤ 70)	132 (58/74)
Elderly (age > 70)	64 (31/33)
Age (years old, mean ± SD)	
All participants	64.33 ± 10.39
Middle-aged	59.43 ± 9.09 (min: 35, max: 70)
Elderly	74.42 ± 2.97 (min: 71, max: 81)

### The intestinal microbiota composition shows differences between the elderly and the middle-aged

**Fig. 2:**
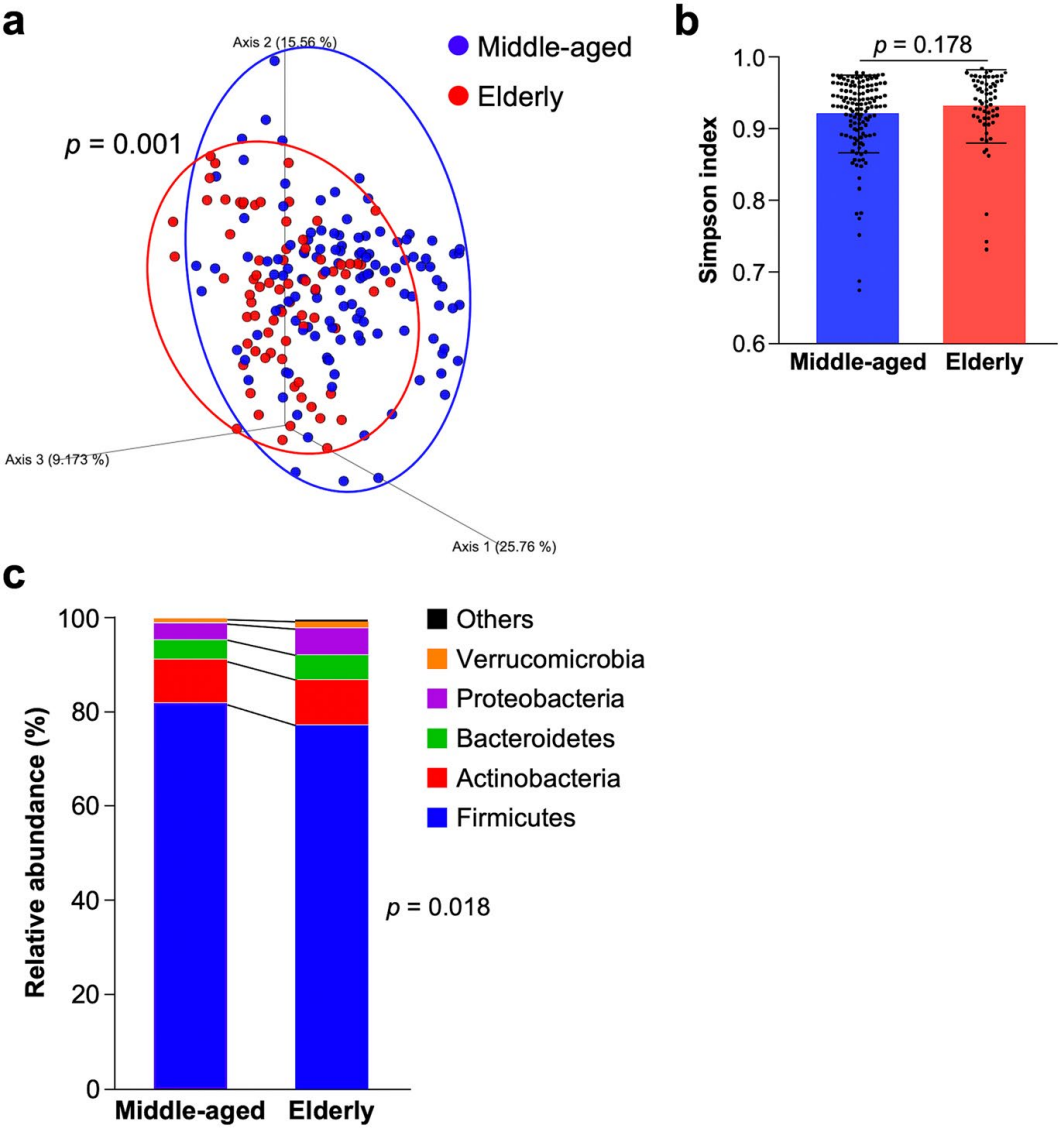
Overall structure of the intestinal microbiota differs between elderly people and middle-aged. **a** β-Diversity analysis by principal coordinate analysis plot based weighted UniFrac distance. **b** α-Diversity analysis by Simpson index. **c** Stacked bar chart of relative abundance of each taxon at the phylum level. Error bars represent mean ± SD. Statistical significance was evaluated by PERMANOVA in **a** and unpaired Student’s *t*-test in **b** and **c**

Next, to analyze the effects of aging on the intestinal microbiota composition, metagenomic 16S rDNA sequencing was conducted. In β-diversity, the intestinal microbiota composition of the elderly was significantly different from that of the middle-aged (Fig. 2a, *p* = 0,001). In contrast, Simpson index, an indicator of α-diversity, showed no significant difference between the two groups (Fig. 2b, middle-aged vs elderly: 0.92 ± 0.05 vs 0.93 ± 0.05, *p* = 0.178). In the analysis of the intestinal microbiota composition at the phylum level, the relative abundance of Firmicutes in the elderly was significantly lower compared to the middle aged (Fig. 2c, middle-aged vs elderly: 80.64 ± 12.89 vs 77.48 ± 13.09%, *p* = 0.018). Bacteroidetes (middle-aged vs elderly: 4.18 ± 5.00% vs 5.31 ± 4.80%, *p* = 0.135) and Proteobacteria (middle-aged vs elderly: 3.51 ± 8.14% vs 5.62 ± 10.02%, *p* = 0.117) showed a tendency of higher in the elderly compared to the middle-aged. Next, to reveal the details of the differences in the intestinal microbiota between the elderly and the middle-aged, relative abundance at the genus level was analyzed (Table 2). In the elderly, relative abundance of *Collinsella*, *Alistipes*, Peptococcaceae; unassigned, *Lactobacillus*, *Lactococcus*, *Weissella*, *Christensenellaceae R-7 group*, *Megasphaera*, and *[Eubacterium] eligens group* were significantly higher and Lachnospiraceae; unassigned, *Blautia*, *Anaerostipes*, *Fusicatenibacter*, *Dorea*, and *Faecalibacterium* were significantly lower compared to the middle-aged. These results indicate that the intestinal microbiota composition of the participants in this study shows age-related differences.

**Table 2:** Relative abundance of significantly differed genera between middle-aged and elderly-group

Taxon					Relative abundance (%, mean ± SD)		
Phylum	Class	Order	Family	Genus	Middle-aged	Elderly	*p* value
Actinobacteria	Coriobacteriia	Coriobacteriales	Coriobacteriaceae	*Collinsella*	0.46 ± 1.35	1.43 ± 1.81	< 0.001
Bacteroidetes	Bacteroidia	Bacteroidales	Rikenellaceae	*Alistipes*	0.27 ± 0.52	0.55 ± 0.90	0.005
Firmicutes	Clostridia	Clostridiales	Peptostreptococcaceae	Unassigned	2.29 ± 3.79	4.06 ± 5.26	0.008
Firmicutes	Bacilli	Lactobacillales	Lactobacilaceae	*Lactobacillus*	1.79 ± 4.98	3.87 ± 8.32	0.031
Firmicutes	Bacilli	Lactobacillales	Streptcoccaceae	*Lactococcus*	0.12 ± 0.78	0.59 ± 2.20	0.032
Firmicutes	Bacilli	Lactobacillales	Leuconostocaceae	*Weissella*	0.04 ± 0.22	0.50 ± 2.05	0.011
Firmicutes	Clostridia	Clostridiales	Christensenellaceae	*R-7 group*	0.19 ± 0.63	0.64 ± 1.28	0.001
Firmicutes	Negativicutes	Selenomonadales	Veillonellaceae	*Megasphaera*	0.08 ± 0.37	0.59 ± 1.61	0.001
Firmicutes	Clostridia	Clostridiales	Lachnospiraceae	*[Eubacterium] eligens group*	0.06 ± 0.16	0.27 ± 0.62	< 0.001
Firmicutes	Clostridia	Clostridiales	Lachnospiraceae	Unassigned	9.48 ± 7.09	6.67 ± 8.28	0.015
Firmicutes	Clostridia	Clostridiales	Lachnospiraceae	*Blautia*	8.42 ± 6.41	5.82 ± 4.38	0.004
Firmicutes	Clostridia	Clostridiales	Lachnospiraceae	*Anaerostipes*	3.77 ± 4.15	2.52 ± 3.77	0.042
Firmicutes	Clostridia	Clostridiales	Lachnospiraceae	*Fusicatenibacter*	3.77 ± 3.97	2.24 ± 2.40	0.005
Firmicutes	Clostridia	Clostridiales	Lachnospiraceae	*Dorea*	2.61 ± 3.03	1.51 ± 1.94	0.008
Firmicutes	Clostridia	Clostridiales	Ruminococcaceae	*Faecalibacterium*	3.18 ± 3.58	2.21 ± 1.94	0.045

All genera showing significant differences (*p* < 0.05) between the middle-aged and the elderly in unpaired Student’s *t*-test and relative abundance more than 0.1% on average of all participants were presented

### Low human defensin 5 in the elderly correlates with age-related differences in the intestinal microbiota composition

**Fig. 3:**
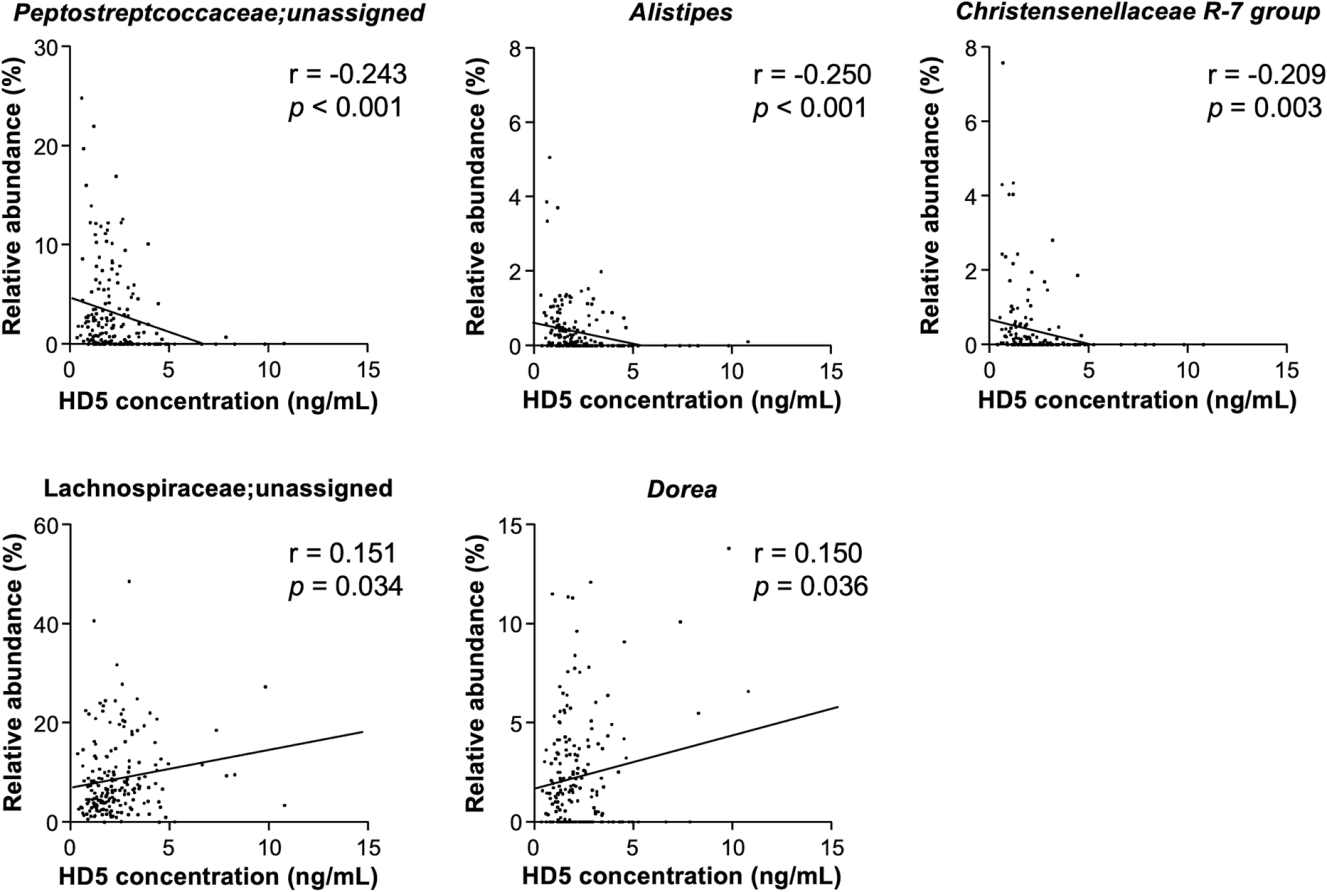
Low HD5 secretion in the elderly correlates with the age-related difference of the intestinal microbiota. Correlation analysis of fecal HD5 concentration and relative abundance of significantly differed genera between the middle-aged and elderly group was conducted. Only the genera showing statistically significant correlation (*p* < 0.05) were presented. Statistical significance was evaluated by Pearson’s correlation coefficients test

Finally, to reveal the relationship between the amount of HD5 secretion and the intestinal microbiota composition in elderly and middle-aged people, correlation analyses between fecal HD5 concentration in each participant and relative abundance of genera showing a significantly different occupancy between the group were conducted (Fig. 3 and Supplementary Table 1). Among the genera that were higher in the elderly compared to the middle-aged, Peptococcaceae; unassigned, *Alistipes*, and *Christensenellaceae R-7 group* showed significant negative correlation against fecal HD5 concentration. In contrast, among the lower genera in the elderly compared to the middle-aged, Lachnospiraceae; unassigned and *Dorea*, showed a significant positive correlation against fecal HD5 level. Thus, it is suggested that lower HD5 secretion in the elderly compared to the middle-aged is associated with the age-related compositional differences of the intestinal microbiota.

## Discussion

It has been reported that the downregulation in immune function both in the innate and acquired immunity termed immunosenescence occurs along with aging [6]. The intestine is the largest immune organ, and the intestinal epithelial cells separating inside and outside of our body play an important role as the front line of intestinal immunity [27]. It is reported that uptake of foreign antigens by M cells in Payer’s patch is suppressed in aged mice [53] and tight junction in small intestinal epithelium is disrupted in aged rats [54], indicating that aging induces a functional decline in small intestinal epithelial cells. Paneth cells, a lineage of small intestinal epithelial cells, play important roles in both innate enteric immunity by secreting α-defensins into the intestinal lumen in response to bacterial, cholinergic, or dietary stimuli and regulation of regeneration and differentiation of small intestinal epithelium by constituting stem cell niche [55, 56]. Furthermore, Paneth cell α-defensins have been known to regulate the intestinal microbiota composition and further contribute to maintaining intestinal homeostasis [57]. Although it has been reported that aging decreases the supply of Wnt signaling to stem cells by Paneth cells [43, 44], the effects of aging on the innate immune function of Paneth cells have been completely unknown. In this study, we revealed that the amount of HD5 secretion in elderly people is lower than middle-aged, illustrating a novel potential mechanism of immunosenescence in innate enteric immunity. This study did not address precise mechanisms that aging induces the decrease of HD5 secretion from Paneth cells, and further future studies to understand the mechanism are needed. To date, it is reported that peripheral blood monocytes obtained from elderly people (age ≥ 65 years old) show reduced pro-inflammatory cytokine production in response to pattern recognition receptor agonist stimulation compared to those from younger adults (age ≤ 40 years old) [58]. Therefore, it may be possible that aging also leads to the decrease in reactivities of Paneth cells against bacterial antigens and further induces the decrease of α-defensin secretion from Paneth cells. Several studies using mouse model revealed that both starvation and a high-fat diet decrease Crps expression [59, 60] and deletion of vitamin D receptor and zinc transporter lead to granule deformity of Paneth cell [61, 62]. In addition, Paneth cells secrete their granules by direct sensing not only bacterial and cholinergic stimuli [33–35] but also certain nutrients or food factors [36], suggesting that food factors regulate Paneth cell α-defensin secretion. Taken together, changes in eating habits along with aging may contribute to the decrease of HD5 secretion in elderly people.

In this study, the relative abundance of Bacteroidetes and Proteobacteria in the elderly had a higher tendency compared to the middle-aged. These trends are consistent with previous cross-sectional studies analyzing age-related changes in the intestinal microbiota composition [22, 23]. In the genus level, relative abundance of Peptostreptococcaceae; unassigned, *Alistipes*, and *Christensenellaceae R-7 group*, which show higher occupancy in the elderly compared to the middle-aged, was negatively correlated, and Lachnospiraceae; unassigned and *Dorea*, which show lower occupancy in the elderly compared to the middle-aged, was positively correlated with the amount of HD5 in feces. These results suggest that decreased secretion of HD5 induces compositional changes in the intestinal microbiota along with aging. Recently, it has been reported that transition of the intestinal microbiota composition is involved in an increased risk of various diseases along with aging [25, 26]. An increase of Peptostreptococcaceae has been reported in patients of NAFLD [63] and obesity [64], suggesting the relationships with lifestyle diseases. It has also been reported that the increase of *Alistipes* relates to certain diseases such as hypertension, colorectal cancer, and depression [65]. Several cohort studies have reported that relative abundance of Christensenellaceae inversely correlates with body mass index (BMI) and increases along with aging [66]. Since it has been known that low BMI increases all-cause mortality risk in the elderly more than high BMI [67, 68], an increase of Christensenellaceae in the elderly may lead to an increased risk of diseases. On the other hand, decreased taxa in the elderly of this study, Lachnospiraceae; unassigned and *Dorea*, both belong to the family of Lachnospiraceae, which is known as the major butylate-producing bacteria in the intestine [69]. Butyric acid produced by the intestinal microbiota is known to induce regulatory T cell differentiation in the intestine [13] and promote neuron proliferation in the hippocampus [70]; thus, the decrease of these bacteria may lead to systemic inflammation and decline of cognitive function along with aging. Our findings in healthy elderly suggest that decreased HD5 along with aging induces compositional changes of the intestinal microbiota and may increase the potential risk of certain age-related diseases.

It has been reported that α-defensin-positive cells appear in the small intestine at embryonic day 15 in mice [71] and gestational age 13 to 16 weeks in humans [72], suggesting that α-defensins contribute to regulating the intestinal environment from the developmental phase of the host. In this study, we showed for the first time that Paneth cell α-defensins continue to be secreted into the intestinal lumen in the elderly, while the secretory amount is lower than the middle-aged. Although the intestinal microbiota composition is known to change along with aging, it has been reported that core microbes of the intestinal microbiota are common from young adults (22 to 48 years old) to semi-supercentenarians (105 to 109 years old) [73]. Taken together, although further studies are needed, it is conceivable that Paneth cell α-defensins contribute to the development and maintenance of the intestinal microbiota throughout humans’ lifetime by continuously secreted into the intestinal lumen from the developmental phase to the elderly.

Recent ex vivo and in vivo studies using mice have reported that α-defensins contribute to not only innate enteric immunity but also the regulation of the intestinal microbiota, and its abnormalities further affect certain disease conditions. It has been reported that the intestinal microbiota of *HD5* transgenic mice shows different composition with increased Bacteroidetes and decreased Firmicutes, whereas knockout mice of *MMP7*, an essential hydrolase for converting inactive pro-form of α-defensins into active mature-form, shows contrary with decreased Bacteroidetes and increased Firmicutes, compared to wild-type mice [37]. Furthermore, mouse Paneth cell α-defensins, Crps with correct folding of intramolecular disulfide bonds, i.e., the oxidized-form Crps, elicit strong bactericidal activities against pathogenic bacteria such as *Salmonella typhimurium* and *Staphylococcus aureus* in vitro, whereas it shows no or minimal bactericidal activities against commensal bacteria such as *Bifidobacterium longum* and *Lactobacillus casei* [38], suggesting that α-defensins secreted into the intestinal lumen regulate the intestinal microbiota composition by eliciting selective bactericidal activities. It has been also reported that the expression level of HD5 is decreased in patients with Crohn’s disease and obesity which are known to relate to dysbiosis [74, 75]. Furthermore, we have revealed that both quantitative and qualitative impairments of α-defensin secretion are associated with pathological progressions of graft versus host disease and Crohn’s disease model mice via dysbiosis [40–42]. Taken together, it is suggested that deficiencies of α-defensin secretion relate to the increased risk of several diseases via inducing dysbiosis. However, comprehensive studies analyzing relationships among Paneth cell α-defensin secretion in humans, the intestinal microbiota, and the disease risk have yet to be conducted. In this study, we suggested that lower HD5 secretion in elderly people relates with increased risk of age-related diseases via dysbiosis by showing the first evidence in human that verify the relationships between Paneth cell α-defensins and the intestinal microbiota, which have been clarified in previous studies using mice and in vitro models. Given the previous studies, it is reasonable to consider the observed differences of the intestinal microbiota in the elderly of this study compared to the middle-aged as the consequence of dysbiosis due to low HD5 secretion. Another possibility for lower HD5 in the elderly is not a causal factor but a result from the compositional differences in the intestinal microbiota compared to the middle-aged. Regarding this possibility, it has been reported that morphological abnormalities in Paneth cells and secretion of misfolded Crps, i.e., dysfunctional reduced-form Crps, occurred in Crohn’s disease model SAMP1/YitFc mice under conventional conditions are also observed after the depletion of all the intestinal microbiota by antibiotic treatments, suggesting disorders in Paneth cells and α-defensins precede changes of the intestinal microbiota [41]. Although further studies are required, we conclude that the compositional differences of the intestinal microbiota were at least partially related to the low amount of HD5 secretion. Importantly, this study demonstrates that low secretion of HD5 partially correlated with the compositional differences of the intestinal microbiota between the elderly and the middle-aged, suggesting Paneth cell α-defensins are one of the key regulators in the age-related transition of the intestinal microbiota composition. Our findings add a novel insight into developing preventives and therapeutics for age-related diseases through the restoration of the intestinal microbiota homeostasis by normalizing the amounts of Paneth cell α-defensins in the intestine. We reported previously that butyric acid, known as a food factor and metabolite of the intestinal microbiota, and leucine, an amino acid obtained from foods, induce secretion of Paneth cell α-defensins [36]. Future comprehensive studies for exploring the enhancer of α-defensin secretion including granule secretion analyses of Paneth cells using enteroid, an ex vivo culture system of small intestinal epithelium [34], oral administration experiments using mouse models, and clinical trials will lead to the discovery of novel functional agents in maintaining health and anti-aging medicine targeting innate enteric immunity and the intestinal microbiota.

## Supplementary Information

Below is the link to the electronic supplementary material.

Supplementary file1 (DOCX 5166 KB)

## Data Availability

The dataset supporting the current study has not been deposited in a public repository to keep participant data secure but is available from the corresponding author on request.
