# Supplementary material for: Lower human defensin 5 in elderly people compared to middle-aged is associated with differences in the intestinal microbiota composition: the DOSANCO Health Study
Source: GeroScience. 2021 Jun 8;44(2):997–1009. doi: 10.1007/s11357-021-00398-y (PMC9135951; doi:10.1007/s11357-021-00398-y)
Supplement: Supplementary file 1 — Supplementary file1 (DOCX 5166 KB) [file 11357_2021_398_MOESM1_ESM.docx]

**Supplementary Information**

**Lower human defensin 5 in elderly people compared to middle-aged is associated with differences in the intestinal microbiota composition: the DOSANCO Health Study**

Yu Shimizu (ORCID ID: 0000-0002-5439-7678)^1^, Kiminori Nakamura (ORCID ID: 0000-0002-2138-1733)^1^, Mani Kikuchi (ORCID ID: 0000-0003-1144-4873)^1^, Shigekazu Ukawa (ORCID ID: 0000-0003-4084-1720)^2^, Koshi Nakamura (ORCID ID: 0000-0001-8141-7614)^3^, Emiko Okada (ORCID ID: 0000-0002-9345-0937)^4^, Akihiro Imae^5^, Takafumi Nakagawa^6^, Ryodai Yamamura (ORCID ID: 0000-0002-9342-7702)^7^, Akiko Tamakoshi (ORCID ID: 0000-0002-9761-3879)^8^, Tokiyoshi Ayabe (ORCID ID: 0000-0002-8631-5593)^1^

^1^Department of Cell Biological Science, Faculty of Advanced Life Science, Hokkaido University, North 21, West 11, Kita-ku, Sapporo, Hokkaido, 001-0021, Japan

^2^Reserch Unit of Advanced Interdisciplinary Care Science, Osaka City University Graduate School of Human Life Science, 3-3-138, Sugimoto, Sumiyoshi-ku, Osaka, 558-8585, Japan

^3^Department of Public Health and Hygiene, Graduate School of Medicine, University of the Ryukyus, 207, Uehara, Nishihara-cho, Okinawa, 903-0215, Japan

^4^Department of Nutritional Epidemiology and Shokuiku, National Institute of Biomedical Innovation, Health and Nutrition, 1-23-1, Toyama, Shinjuku-ku, Tokyo, 162-8636, Japan

^5^Suttu Municipal Clinic, 72-2, Toshimacho, Suttu-cho, Hokkaido, 048-0406, Japan

^6^The Centre of Family Medicine, 1-18, North 41, East 15, Higashi-ku, Sapporo, Hokkaido, 007-0841, Japan

^7^Division of Biomedical Oncology, Institute for Genetic Medicine, Hokkaido University, North 15, West 7, Kita-ku, Sapporo, Hokkaido, 060-0815, Japan

^8^Department of Public Health, Faculty of Medicine, Hokkaido University, North 15, West 7, Kita-ku, Sapporo, Hokkaido, 060-8638, Japan

Correspondence: Tokiyoshi Ayabe, M.D., Ph.D.

Professor

E-mail: ayabe@sci.hokudai.ac.jp

**
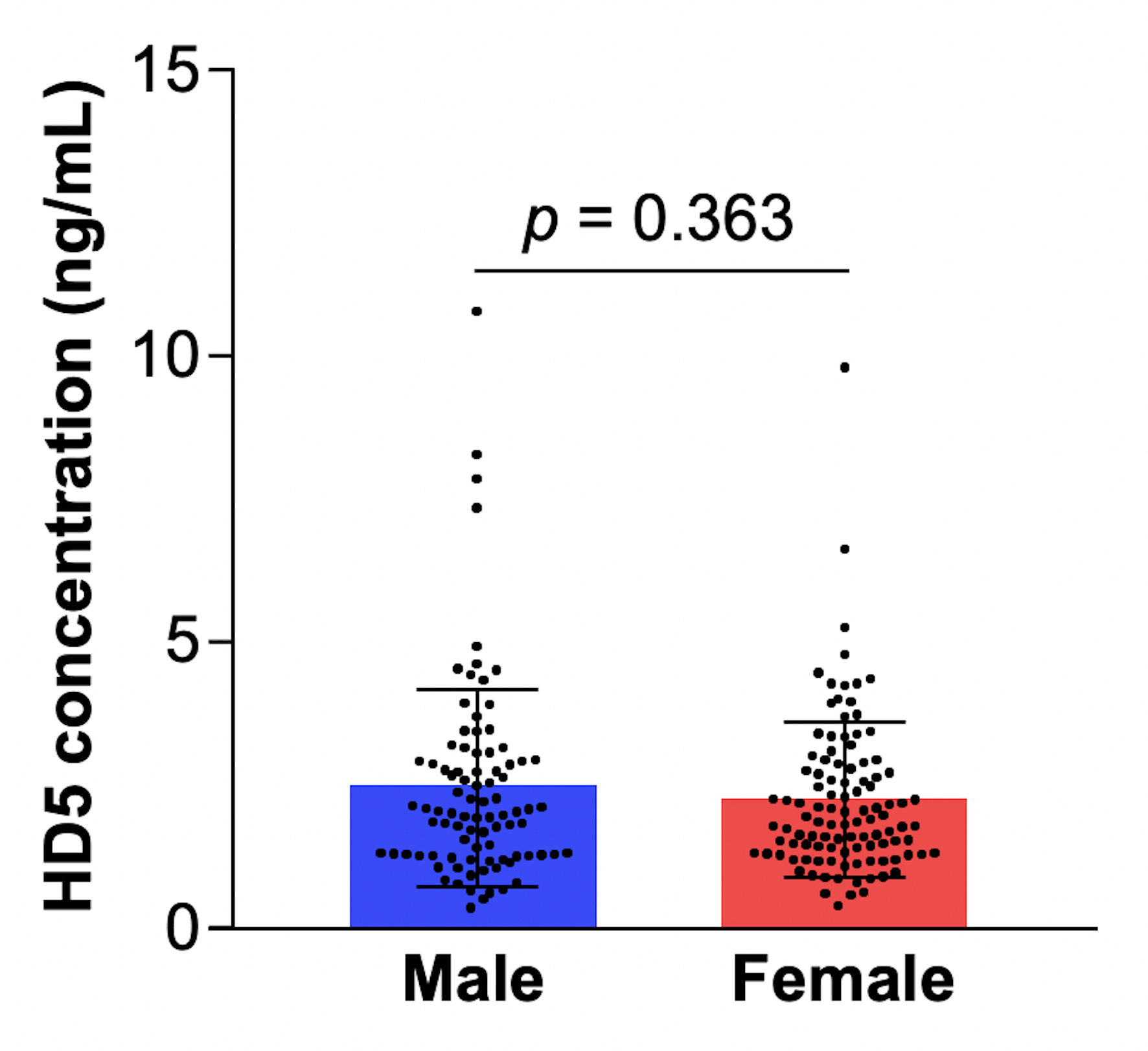
**

**Supplementary Fig. 1** Comparison of fecal HD5 concentration between male and female participants. Error bars represent mean ± SD. Statistical significance was evaluated by unpaired Student’s *t*-test

**Supplementary Table 1** Full list of correlation analysis between fecal HD5 concentration and relative abundance of significantly differed genera between middle-aged and elderly group


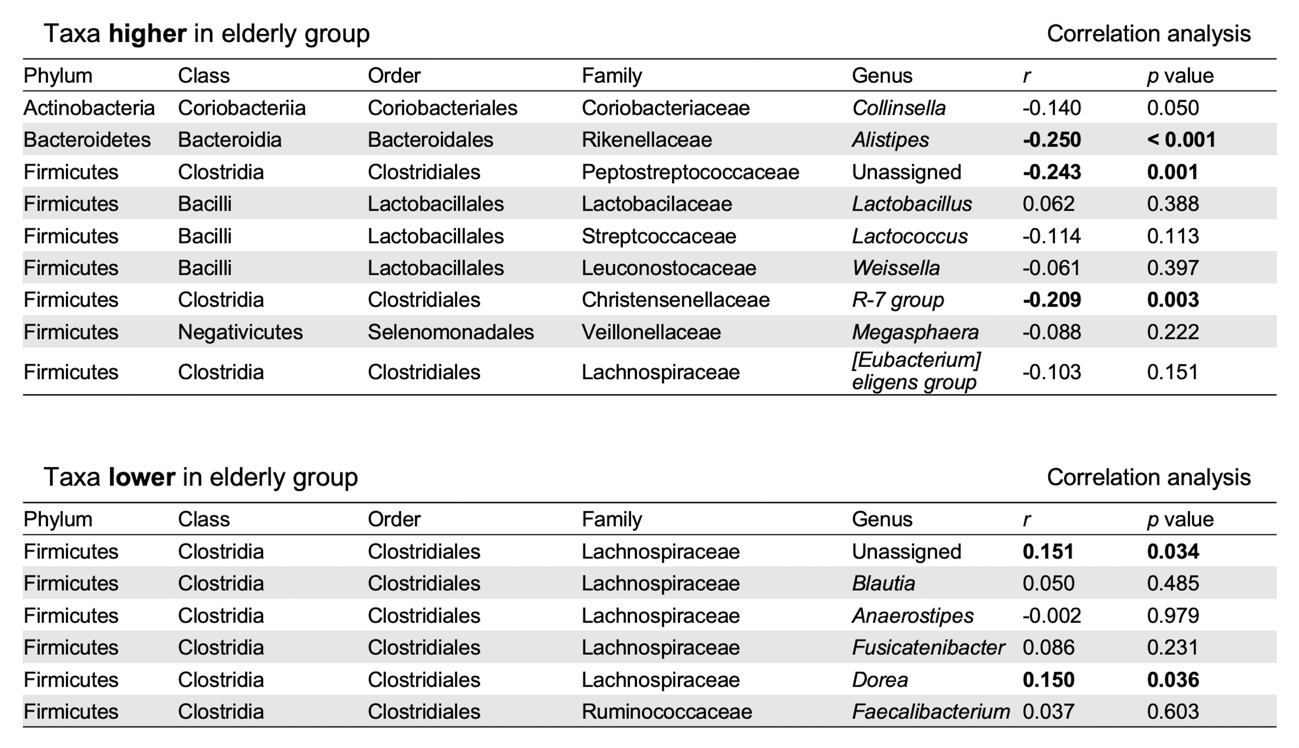


Statistical significance was evaluated by Pearson’s correlation coefficient test. Bold fonts mean statistically significant (*p* < 0.05)
